# Major clinical benefit from adjuvant chemotherapy for stage II–III non-small cell lung cancer patients aged 75 years or older: a propensity score-matched analysis

**DOI:** 10.1186/s12890-022-02043-6

**Published:** 2022-06-28

**Authors:** Miriam Blasi, Martin E. Eichhorn, Petros Christopoulos, Hauke Winter, Claus Peter Heußel, Felix J. Herth, Rami El Shafie, Katharina Kriegsmann, Mark Kriegsmann, Albrecht Stenzinger, Helge Bischoff, Michael Thomas, Jonas Kuon

**Affiliations:** 1 Department of Thoracic Oncology, Thoraxklinik at Heidelberg University Hospital, Röntgenstraße 1, 69126 Heidelberg, Germany; 2 Translational Lung Research Center (TLRC) Heidelberg, Member of the German Center for Lung Research (DZL), Heidelberg, Germany; 3 Department of Thoracic Surgery, Thoraxklinik at Heidelberg University Hospital, Heidelberg, Germany; 4 Department of Diagnostic and Interventional Radiology With Nuclear Medicine, Thoraxklinik at Heidelberg University Hospital, Heidelberg, Germany; 5 Department of Pneumology, Thoraxklinik at Heidelberg University Hospital, Heidelberg, Germany; 6 Department of Radiation Oncology, Heidelberg University Hospital, Heidelberg, Germany; 7 Department of Hematology, Oncology and Rheumatology, Heidelberg University Hospital, Heidelberg, Germany; 8 Institute of Pathology, Heidelberg University Hospital, Heidelberg, Germany; 9 Department of Diagnostic and Interventional Radiology, University Hospital, Heidelberg, Germany

**Keywords:** Adjuvant chemotherapy, Elderly, Non-small cell lung cancer

## Abstract

**Background:**

Data are currently insufficient to support the use of adjuvant chemotherapy (ACT) after surgical resection for stage II or III non-small cell lung cancer (NSCLC) in patients aged ≥ 75 years. In this study we evaluated efficacy and safety profile of ACT in this population.

**Methods:**

We retrospectively evaluated 140 patients ≥ 75 years who underwent curative surgical resection for stage II–III NSCLC from 2010 to 2018 with an indication to ACT according to current guidelines. A propensity score-matched analysis was performed to avoid cofounding biases.

**Results:**

Thirty of 140 patients (21%) received ACT. Most patients (n = 24, 80%) received carboplatin in combination with vinorelbine, while 5 patients (17%) received cisplatin plus vinorelbine and one patient (3%) carboplatin plus gemcitabine. The occurrence of adverse events led to treatment discontinuation in 8 (27%) cases, while 19 (63%) patients completed 4 chemotherapy cycles. Common reported adverse events with ACT were anemia (n = 20, 67%), neutropenia (n = 18, 60%), thrombocytopenia (n = 9, 30%), renal impairment (n = 4, 13%) and transaminase elevation (n = 4, 13%). No toxic deaths occurred. The median follow-up was 67 months (IQR: 53–87). ACT was associated with a significant benefit in both relapse-free survival (median 36 vs. 18.5 months, p = 0.049) and overall survival (median not reached [NR] vs. 33.5 months, p = 0.023) in a propensity score-matched analysis which controlled for cofounders.

**Conclusion:**

ACT confers a survival benefit after curative resection of stage II–III NSCLC in selected patients aged 75 years or older with a manageable toxicity profile.

**Supplementary Information:**

The online version contains supplementary material available at 10.1186/s12890-022-02043-6.

## Background

Lung cancer, the leading cause of death from cancer worldwide, is a disease of older adults [1]: the median age at the diagnosis is 70 years and approximately 37% of the patients are older than 75 years [2]. Non-small-cell lung cancer (NSCLC) patients diagnosed with stage I to IIIA disease are eligible for surgical resection with curative intent. However, 35% of these patients die because of local or metastatic tumor recurrence, which occurs in 65–75% of the patients [3].

The eighth edition of the TNM lung cancer staging system proposed by the International Association for the Study of Lung Cancer (IASLC) [4] is currently used. Discrepancies exist between the 7th TNM staging system and 8th TNM staging system: node-negative tumors > 4 cm but ≤ 5 cm have been classified as stage IIA instead of stage IB in 8th TNM staging system and the classification of T3 and T4 tumors has changed, as well as those of stage III subgroups. For this reason, many results from previous studies may not be directly translated into current clinical practice.

Surgery followed by adjuvant chemotherapy for patients with stage II–IIIA NSCLC represents the standard of care. Several phase III randomized controlled trials demonstrated the associated survival benefit [5–7], corresponding to a 5-year absolute overall survival (OS) benefit of 5.4% [8]. Despite the advent of immune-oncology and tyrosine-kinase inhibitors in the treatment of advanced and locally advanced NSCLC, the standard of care in the adjuvant setting is still platinum-based chemotherapy in the vast majority of cases. Only very recently osimertinib has been approved in the adjuvant setting in patients with stage IB-IIIA NSCLC harboring EGFR exon 19 deletions or exon 21 L858R substitution mutations [9].

The Lung Adjuvant Cisplatin Evaluation (LACE) pooled analysis showed a negative effect on survival outcomes of adjuvant chemotherapy for stage IA, while the risk reduction for death was 8% for stage IB [8]. Other studies have shown no clear survival advantage for adjuvant chemotherapy among patients operated for stage IB NSCLC, while a subgroup analysis from the Cancer and Leukemia Group B (CALGB) 9633 trial reported a statistically significant survival benefit with adjuvant chemotherapy for tumors ≥ 4 cm [10] (stage II in the TNM 8th edition). For these reasons adjuvant chemotherapy has been debated for patients with stage IB tumors, while it has been considered as standard of care for tumors ≥ 4 cm [11] and most of the trials have included patients with stage IB ≥ 4 (stage II in the TNM 8th edition) cm in addition to stage II and IIIA.

On the other hand, optimal management of patients with stage III disease remains challenging in the context of highly heterogeneous disease characteristics that require multidisciplinary approaches.

There is a lack of evidence regarding adjuvant chemotherapy in older patients, which translates into the absence of clear guidelines for the use of adjuvant treatment in this population. This is due to the lack of large prospective studies specifically testing adjuvant chemotherapy in older patients and the absence of a representative population in main clinical trials: only the 9% of patients in a meta-analysis of randomized trials were older than 70 years [12]. Our knowledge is limited to retrospective studies of population databases and post hoc analyses of prospective studies.

However, despite the utilization of dosage adjustments, the lower dose intensity and the use of carboplatin instead of cisplatin, the elderly population has been shown to experience survival benefit from adjuvant treatment [13, 14]. Moreover, no differences in severe toxicity rates were found when patients over 65 years old were compared to younger patients in a LACE pooled analysis [12]. However, the survival benefit was limited to patients aged less than 80 for stage II–IIIA [15] and to patients aged less than 75 for stage IB ≥ 4 cm [3], although these findings should be considered with caution since they originate from post hoc analyses with small sample sizes, and adjuvant chemotherapy is not recommended for patients ≥ 75 years old in the guidelines [11].

In spite of the reported survival gain, the similar tolerability to those of younger patients and the possibility to use carboplatin without a survival disadvantage [15], adjuvant chemotherapy is still not offered to the vast majority of older adults, mostly due to the lack of a proven benefit.

The purpose of this retrospective study was to identify the role of adjuvant chemotherapy and to collect real-world data on toxicity rates, dose adjustments and dose intensity in patients aged 75 years or older that underwent curative surgical resection for NSCLC.

## Materials and methods

### Patient cohort

We conducted a monocentric, retrospective review of patients aged 75 years or older that underwent curative surgical resection for stage II–IIIA NSCLC in accordance with the 8th edition of the TNM classification at the Thoraxklinik Heidelberg from 2010 to 2018. Although T3N2M0 and T4N2M0 tumors have been reclassified to stage IIIB in the 8th edition of the IASLC staging system, these patients remain eligible (as stage IIIA under the 7th edition criteria). Patients with performance status (PS) according to Eastern Cooperative Oncology Group (ECOG) ≤ 1, R0 surgery, glomerular filtration rate ≥ 45 ml/min and survival from the surgical intervention of at least 30 days were included.

We retrospectively collected clinicopathological features such as gender, age at the time of surgery, comorbidities, PS ECOG, renal function at the time of surgery, histology, tumor (T) and nodal (N) status, stage of the tumor, chemotherapy dosing and dose adjustments and discontinuations. Adverse events were recorded after every cycle of chemotherapy and graded according to the National Cancer Institute Common Terminology Criteria for Adverse Events version 5.0 (CTCAE 5.0). Comorbidities were summarized using Charlson Comorbidity Index (CCI). We did not include lung cancer as a comorbidity in the scores’ calculation. We also reviewed follow-up data of tumor recurrence and death.

### Statistical analysis

We used descriptive statistics to summarize patients’ characteristics and toxicity profile. Results are presented as absolute numbers and percentages and median (minimum [min.]–maximum [max.]), where appropriate. Chi-square and Fisher’s exact test were used to compare categorical variables between observation and chemotherapy group. Between-group comparisons of continuous variables were performed with Mann–Whitney test.

Univariate logistic regression was used to (retrospectively) identify factors associated with the treating physician’s choice to administer adjuvant chemotherapy or not (binary dependent variable). Gender, age, T status, N status, stage of the tumor, PS ECOG, CCI were considered as independent variables. Results are reported as Odds ratios (OR) and 95% confidence intervals (CI).

We performed a 1:1 propensity score matching to compare patients based on adjuvant chemotherapy receipt. The matching was done based on age, stage and N-status. We matched 30 patients treated with adjuvant chemotherapy with one patient each from the observation group based on the above-mentioned matching criteria. Survival analysis were performed on the propensity-matched sample.

The relapse-free survival period (RFS) was defined as the time from the date of the surgery to the date of recurrence or death or to the last date in which the patient was known to be disease-free. The overall survival (OS) period was defined as the time from the date of the surgery to the day of death or the last date in which the patient was known to be alive. RFS and OS were assessed using the Kaplan–Meier method and the differences between the curves were tested using the two-tailed log-rank test.

As sensitivity analysis, univariable and multivariable Cox regression hazard models were applied to the entire (unmatched) population including all potentially relevant clinical variables, like post-surgical treatment (observation/adjuvant chemotherapy), age group (< 80/ ≥ 80), sex, ECOG score, CCI (0–1/ ≥ 2), histology, smoking history, N-stage, T-stage, type of surgical operation (lobar resection/sub-lobar resection/pneumonectomy) and pathologic stage, in order to identify parameters significantly associated with RFS and OS. Variables with p < 0.2 in the univariable testing were subsequently included in the multivariable analysis.

We considered a p-value < 0.05 as statistically significant. All data were analyzed using R version 3.6.2 statistical software (R Foundation for Statistical Computing, Vienna, Austria) with the “survival” (v.3.2–13) and the “MatchIt” packages (v. 4.2.0).

## Results

### Patients´ demographics

Between 2010 and 2018, 259 patients aged 75 years or older underwent surgical resection for lung cancer in our center. We identified 204 patients that underwent surgical resection for early-stage NSCLC with a formal indication to adjuvant chemotherapy according to the guidelines. Of these, 64 patients did not meet the inclusion criteria.

The majority of patients were men (59%) and the median age was 78 years (min.–max.: 75–91).

**Fig. 1:**
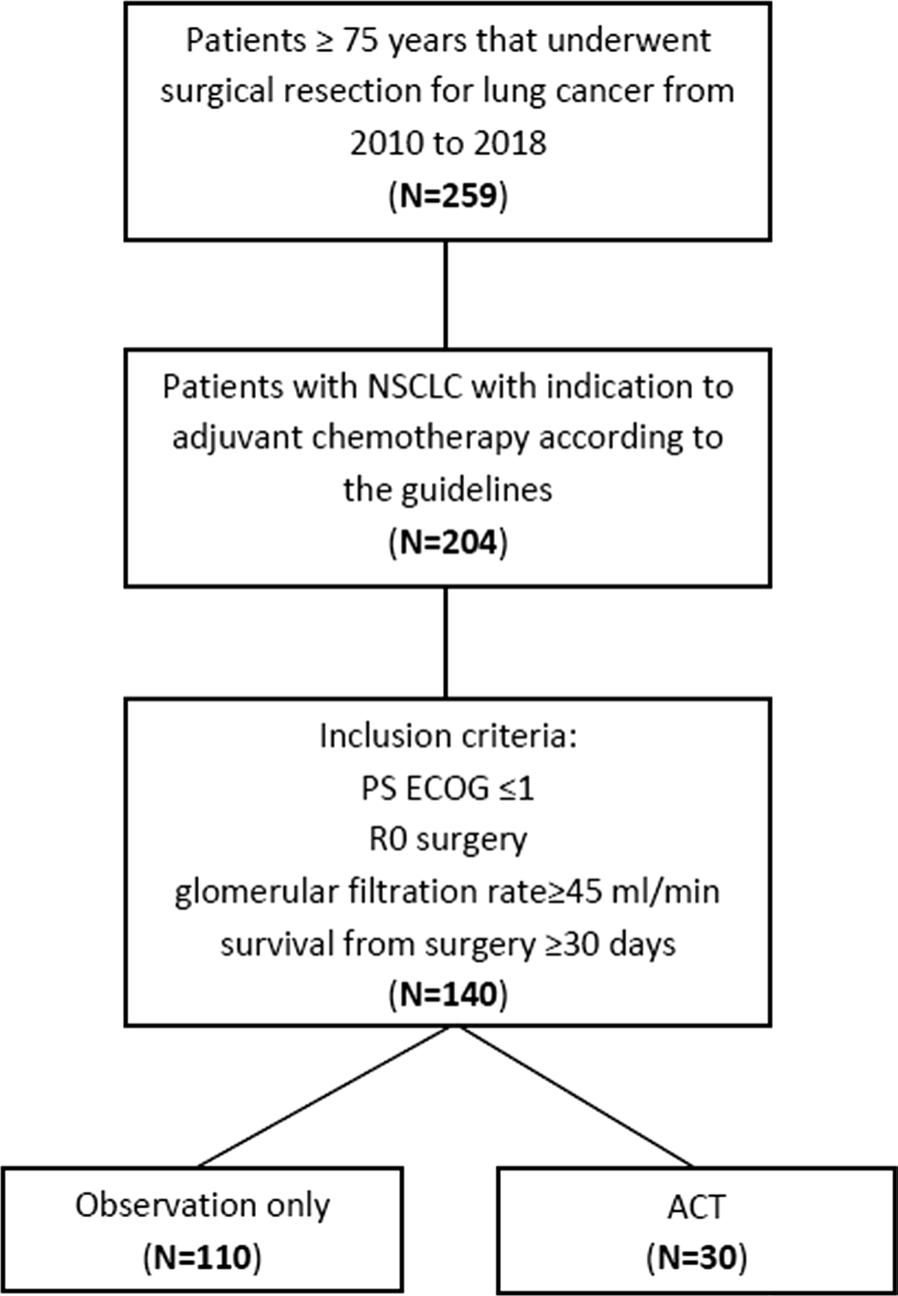
Flowchart of patient cohort selection

Among the 140 included patients, only 30 patients (21%) received adjuvant chemotherapy (Fig. 1). Univariate logistic regression analysis showed that factors significantly associated with receiving adjuvant chemotherapy after curative surgery were age (OR 0.63, 95% CI 0.48–0.79, p < 0.001), N status (OR 2.68, 95% CI 1.53–4.94, p < 0.001) and stage (OR 6.96, 95% CI 2.29–30.39, p = 0.002), while T status, gender, PS ECOG, CCI were not significantly associated with the administration of adjuvant treatment. Patients’ characteristics are provided in Table 1.

**Table 1:** Patients’ characteristics

Variable	Adjuvant chemotherapy	Observation	p-value	Matched observation group	p-value
Patients, n (%)	30 (100)	110 (100)		30 (100)	
Age, years			< 0.001		0.8
Median	76.2	79		76.7	
Min.–Max.	75–81	75–91		75–81	
Gender, n (%)			0.084		0.58
Male	22 (73)	59 (54)		19 (63)	
Female	8 (27)	51 (46)		11 (37)	
Smoking status, n (%)			0.364		0.22
Never	6 (20)	32 (29)		3 (10)	
Former	20 (67)	56 (51)		18 (60)	
Current	4 (13)	22 (20)		9 (30)	
CCI, n (%)			0.46		0.434
0	7 (23)	21 (19)		5 (17)	
1	11(37)	31 (28)		8 (27)	
≥ 2	12 (40)	58 (53)		17 (56)	
Stage, n (%)			0.001		1
II	3 (10)	48 (44)		3 (10)	
III	27 (90)	62 (56)		27 (90)	
T status, n (%)			0.44		0.386
1	1 (3)	2 (2)		2 (7)	
2	11 (37)	37 (34)		7 (23)	
3	7 (23)	43 (39)		11 (37)	
4	11 (37)	28 (25)		10 (33)	
N status, n (%)			< 0.001		0.406
0	6 (20)	43 (39)		7 (23)	
1	8 (27)	48 (44)		12 (40)	
2	16 (53)	19 (17)		11 (37)	
PS ECOG, n (%)			0.206		0.3
0	19 (63)	53 (48)		14 (47)	
1	11 (37)	57 (52)		16 (53)	
Histopathology, n (%)			1		1
Squamous	15 (50)	55 (50)		16 (53)	
Non-squamous	15 (50)	55 (50)		14 (47)	
Type of surgery, n (%)			0.568		0.67
Lobar resection	23 (76)	89 (81)		22 (74)	
Sub-lobar resection	5 (17)	11 (10)		4 (13)	
Pneumonectomy	2 (7)	10 (9)		4 (13)	

CCI, Charlson Comorbidity Index; Max., Maximum; Min., Minimum; PS ECOG, performance status according to Eastern Cooperative Oncology Group, T, tumor

### Chemotherapy dosing, discontinuation rates and toxicity

Among patients treated with adjuvant chemotherapy, 24 (80%) received carboplatin in association with vinorelbine, 5 (17%) cisplatin in association with vinorelbine and one (3%) carboplatin in association with gemcitabine. In total, 8 patients (27%) required treatment discontinuation due to toxicity, while most of the patients (n = 19, 63%) completed all four planned cycles of chemotherapy.

Information on chemotherapy dosage and cycles delays in one patient was missing. Fourteen patients (47%) required dose reduction, 7 patients (23%) from the first cycle and the remaining patients due to the occurrence of toxicity. In 14 patients (47%) chemotherapy was postponed once or more often because of adverse events. Four patients receiving cisplatin required delays of the treatment and three of them required discontinuation for intolerable adverse events.

Information on specific toxicity in four patients was missing. Common reported adverse events were anemia (n = 20, 67%), which reached grade 3 in two cases (7%), and neutropenia (18 patients, 60%), that reached grade 3 in 9 (50%) cases. Granulocyte-colony stimulating factors were administered in 9 patients (30%), 5 patients (17%) experienced febrile neutropenia. Other frequent reported adverse events were thrombocytopenia (n = 9, 30%), renal impairment (n = 4, 13%) and transaminase elevation (n = 4, 13%). No toxicity-related deaths were reported. Chemotherapy regimens and toxicity profile are summarized in Table 2.

**Table 2:** Adjuvant chemotherapy regimens and toxicity profile

	N (%)
Regimen	
Carboplatin-vinorelbine	24 (80)
Cisplatin-vinorelbine	5 (17)
Carboplatin-gemcitabine	1 (3)
Number of cycles	
1	6 (20)
2	2 (7)
3	3 (10)
4	19 (63)
Discontinuation	8 (27)
Treatment-related AEs^1^	
Anemia	20 (67)
Neutropenia	18 (60)
Febrile neutropenia	5 (17)
Thrombocytopenia	9 (30)
Creatinine increase	4 (13)
Transaminase increase	4 (13)

AEs, adverse events

^1^Detailed information about treatment-related AEs missing for 4 patients

### Survival analysis

A total of 30 propensity-matched pairs—one each from the adjuvant chemotherapy and the observation group—were included for this analysis. The groups did not differ significantly in baseline characteristics (Table 1). The median follow-up was 67 months (interquartile range [IQR] 53–87).

The median RFS was 36 months in the chemotherapy group and 18.5 months in the observation group (HR 0.54 [95% confidence interval (CI) 0.28–1], p = 0.049). The 5-year RFS was 46% and 17% in the chemotherapy and the observation group, respectively (p = 0.049).

The median OS was not reached (NR) in patients receiving adjuvant chemotherapy, while it was 33.5 months in patients in the observation group (HR 0.45 [95% CI 0.22–0.92], p = 0.023). The 5-year OS was 61% for the chemotherapy group and 39% for the observation group (p = 0.046).

**Fig. 2:**
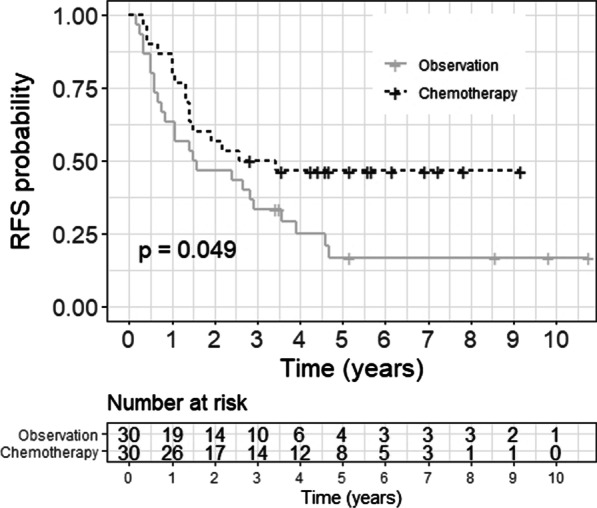
Kaplan–Meier analysis of RFS

**Fig. 3:**
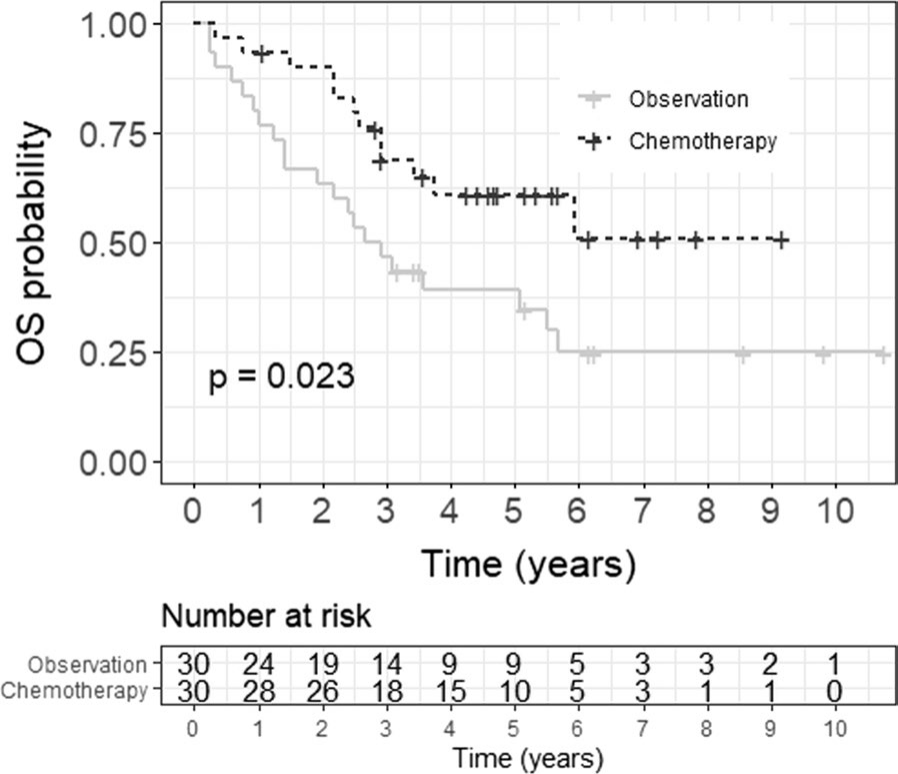
Kaplan–Meier analysis of OS

The Kaplan–Meier curves are shown in Figs. 2 and 3.

The univariable and multivariable Cox proportional hazard models, which were performed for the entire (unmatched) population as sensitivity analyses, are provided as supplementary material (Additional file 1). In the multivariable analysis, adjuvant chemotherapy was identified as an independent and significant factor for RFS (HR 0.49 [95% CI 0.28–0.87], p = 0.015) and OS (HR 0.43 [95% CI 0.22–0.82], p = 0.010) along with ECOG PS (RFS HR 1.66 [95% CI 1.09–2.55], p = 0.020; OS HR 1.84 [95% CI 1.15–2.95], p = 0.011), while pathological stage and nodal status did not show significant results.

## Discussion

In this study we retrospectively evaluated the safety and efficacy profile of adjuvant chemotherapy in selected patients aged 75 years or older that underwent surgical resection for stage II–III NSCLC.

Among patients treated with surgery with curative intent, we selected those eligible for adjuvant chemotherapy for our retrospective analysis. We excluded patients with PS ECOG higher or equal to 2 and moderate to severe renal impairment and those who underwent R1 or R2 surgery or who died within 30 days from surgery. Only 30 of the 140 eligible patients received adjuvant treatment. Factors associated with receiving this treatment modality were younger age, nodal status and stage, while T stage and CCI did not influence the clinicians’ decision. As reported by others, older patients receive adjuvant chemotehrapy less often than younger patients, in spite of a similar survival benefit [16]. Moreover, the role of PS [17] as well as nodal status [18] as important factors influencing the treatment decision in patients with resectable NSCLC is well known.

Despite accumulating evidence that older patients, even receiving a lower total chemotherapy dose, benefit from adjuvant treatment with acceptable toxicity [19], this treatment modality is still denied to the vast majority of them only on the basis of age and concerns about their ability to tolerate platinum-related toxicity [20]. Indeed, these patients have often an impaired renal and liver function, as well as a reduced hematopoiesis and poor PS. Comorbidities and polipharmacy increase the likelihood to experience inacceptable toxicity [20].

Information on benefit and toxicity risks of adjuvant chemotherapy in the elderly population is lacking, particularly for patients aged 75 years or older, due to the underrepresentation of this age group in randomized controlled trials as a consequence of the selection of participants on the base of comorbidities, functional status and age. No elderly-specific trials of adjuvant chemotherapy in NSCLC have been conducted so far and a specific standardized tool for risk assessment in older patients undergoing adjuvant treatment after surgical resection is still lacking [21]. Only recently the Cancer Aging Research Group (CARG) score, which predicts grade 3–5 chemotherapy toxicity in older adults aged 65 years or older [22], has been validated in the adjuvant and neoadjuvant setting [23].

A survival benefit for unselected patients aged 65 years or older that received adjuvant chemotherapy for stage II–IIIA NSCLC was demonstrated in an observational cohort study using population-based data. This benefit was, however, not confirmed in patients aged 80 years or older and the administration of platinum-based adjuvant chemotherapy was associated with an increased risk of toxicity requiring hospital admission. This risk was, however, consistent with those reported in younger patients in previous publications [14]. Moreover, retrospective analyses of controlled trials and pooled analysis evidenced a similar survival benefit for fit older patients compared to younger patients [13, 24].

In our population treated with adjuvant chemotherapy, only four patients (13%) received cisplatin, while the rest of the patients received carboplatin. Treatment discontinuation was required in 27% of the patients, including three patients receiving cisplatin, while dose reductions and cycle delays were required in 43% and 47% of the patients, respectively. With carboplatin and appropriate dose reduction, rates of neutropenia (60%), anemia (67%), febrile neutropenia (17%) and thrombocytopenia (30%) were consistent with those of clinical trials enrolling only patients younger than 75 years old [6] or in which the elderly population represented only a small portion of the enrolled patients [13]. Carboplatin is more commonly used in older patients due to its favorable toxicity profile, with a lower infection and emesis rate. Results from retrospective analyses demonstrated a survival benefit in patients treated with carboplatin-based adjuvant chemotherapy over observation, with no significant differences compared to cisplatin-based chemotherapy [15, 25].

In our study cohort, we have found a significant improvement in RFS (median 36 vs 18.5 months) as well as in OS (median NR vs 33.5 months) in patients receiving adjuvant chemotherapy after surgical resection for stage II–III NSCLC compared to those who did not receive postoperative treatment after propensity score matching. The survival advantage conferred by adjuvant chemotherapy was confirmed in a sensitivity analysis with multivariable Cox regression hazard model which was performed for the entire unmatched population. The benefit conferred by adjuvant chemotherapy in both OS and RFS in patients aged 75 years or older has been reported in other retrospective analyses [26]. The magnitude of the benefit in our cohort is superior to those reported in clinical trials, a discrepancy that can be explained with the high percentage of stage III disease in both populations after propensity-score matching (90% stage III vs. 10% stage II). It is indeed known that stage is a strong predictive factor, with stage III patients benefitting the most from adjuvant chemotherapy [27]. Moreover, our population has a selection bias, in which patients have been carefully evaluated initially for surgical eligibility and in a second time as possible candidates for adjuvant chemotherapy.

Our study has some limitations. The study has a small sample size and is retrospective and not randomized. The decision of whether to use adjuvant chemotherapy was made by the treating physicians, depending on patient’s characteristics, clinical history, and recovery after surgery. Moreover, chemotherapy regimens, dosage adjustments and postponements were not standardized. The use of inclusion criteria and propensity score matching, and the data collected from a single center may however reduce the selection bias.

## Conclusions

In conclusion, our study provides further evidence about the benefits in both RFS and OS of adjuvant chemotherapy after curative resection of stage II–III NSCLC in highly selected patients aged 75 years or older. Chronological age is not sufficient to determine whether an old patient could benefit from the standard treatment with acceptable toxicity. The evaluation should include the assessment of functional status, comorbidities, surgical sequelae and consider the patient’s preference. Chemotherapy still represents the standard of care in the adjuvant setting, but with the advent of new, more tolerable agents more and more elderly patients will be eligible for adjuvant treatment in the future, a fact that underlines the importance of including this patient population in clinical trials. Prospective randomized trials and larger cohorts are needed to confirm our findings.

## Supplementary Information

**Additional file 1.** Univariable and multivariable Cox proportional hazard models for RFS and OS applied on the entire (unmatched) population.

## Data Availability

The datasets generated and analyzed during the current study are not publicly available due to privacy concerns but are available from the corresponding author, JK, on reasonable request.

## Abbreviations

ACT: Adjuvant chemotherapy
NSCLC: Non-small cell lung cancer
IASLC: International Association for the Study of Lung Cancer
OS: Overall survival
LACE: Lung adjuvant cisplatin evaluation
CALGB: Cancer and Leukemia Group B
PS: Performance status
ECOG: Eastern Cooperative Oncology Group
CTCAE: National Cancer Institute Common Terminology Criteria for Adverse Events
CCI: Charlson Comorbidity Index
IQR: Interquartile range
RFS: Relapse free survival
NR: Not reached
CARG: Cancer Aging Research Group
